# Challenges in opioid therapy implementation: national survey of palliative care consultation services

**DOI:** 10.1186/s12904-025-01921-0

**Published:** 2025-10-20

**Authors:** Evelyn Mueller, Susanne Gahr, Annette Schnell, Eva Schildmann, Christopher Boehlke, Carmen Roch

**Affiliations:** 1 Interdisciplinary Center for Palliative Medicine, Department of Radiation Oncology, Comprehensive Cancer Center Mainfranken, University Hospital Wuerzburg, Josef-Schneider-Str. 11, Würzburg, 97080 Germany; 2 Department of Palliative Medicine, CCC Erlangen-EMN, University Hospital Erlangen, Friedrich-Alexander-University Erlangen-Nürnberg (FAU), Universitaetsstr. 21/23, Erlangen, 91054 Germany; 3 Center for Palliative Medicine, CCC Regensburg, University Hospital Regensburg, Franz- Josef-Strauß-Allee 11, Regensburg, 93053 Germany; 4 Palliative Medicine, Faculty of Medicine, University of Augsburg, Stenglinstr. 2, 86156 Augsburg, Germany; 5 Department of Clinical Research, University Hospital Basel and University of Basel, Petersgraben 4, Basel, 4031 Switzerland; 6 Palliative Care Center Bethesda Hospital, Gellertstr. 144, Basel, 4052 Switzerland; 7 Comprehensive Cancer Center Alliance WERA, Bavaria, Germany

**Keywords:** Analgesics, Opioid, Palliative care, Palliative medicine, Consultation

## Abstract

**Background:**

Palliative care consultation services in hospitals can improve symptom control in patients with advanced illness by recommending or prescribing WHO step III opioids. However, effective treatment depends on the attending ward team which implements these therapies. While deviations from the opioid therapy recommended by the palliative care service and also treatment errors are often an issue in everyday life, there is no current data on the extent of the problem. This study explored the experiences of palliative care consultation services with the implementation of opioid recommendations by the attending ward team.

**Methods:**

The questionnaire was developed through a multi-step process, including e.g. literature analysis and pre-testing with cognitive interviews. A closed national online survey was conducted via the SoSci Survey platform. All palliative care consultation services registered with the German Association for Palliative Medicine were invited to participate in November 2024, with a reminder sent three weeks later.

**Results:**

The survey was fully completed by 39 of 85 consultation services (response rate: 46%; 21 university, 18 general hospitals). Thirty-one consultation services provide recommendations for opioids, eight provide prescriptions. Most (23 of 39) reported a rather high or very high need for improvement in implementing opioid recommendations (4-point Likert-scale, very low – very high). Common deviations included “no implementation at all,” “lower dose,” and “non-implementation of medications to prevent side effects.” “Inexperience or reservations about opioid therapy among attending ward staff” was the most frequently cited reason, named by 35 of 39 palliative care consultation services as occurring “sometimes” or “often” (5-point Likert-scale, “(almost) never” – “(almost) always”). Participants highlighted need for improvement in regard to symptom assessment, prescribing and use of PRN (pro re nata; on demand) medication, continuity of opioid therapy at discharge, and opioid treatment in the dying phase. Consultation services providing opioid recommendations reported significantly more frequent deviations from recommendations and a greater need for improvement compared to those prescribing opioids.

**Conclusion:**

Despite a 46% response rate, limiting generalizability, the frequent reporting of collaboration challenges between palliative care consultation services and attending ward teams regarding opioid-based symptom control highlights a relevant problem. Targeted training could improve implementation and symptom management.

**Supplementary Information:**

The online version contains supplementary material available at 10.1186/s12904-025-01921-0.

## Background

WHO step III opioids are frequently recommended by palliative care consultation services (PCCS) in hospitals. There is strong evidence supporting their use in managing pain in patients with advanced cancer [1], heart failure [2] liver and kidney diseases [3]. They are also frequently applied in managing dyspnea, though evidence for their use in this context remains limited and varies by underlying condition [4–6]. 

Concerns about opioid-related harms—such as abuse, addiction, and possible adverse effects—have led to strict prescribing guidelines, which in turn may have contributed to the underuse of opioids in patients with legitimate needs [7, 8]. Studies suggest that around 40% of patients with advanced cancer experience insufficient pain control [9–11]. 

Involving PCCS in the care of patients with advanced illness improves pain management outcomes [12–16]. Most PCCS function as consultation services, visiting patients with complex symptom needs upon request and providing recommendations to attending physicians. The attending physicians and nurses are then required to prescribe and administer the recommended drugs [17–19]. 

Data on implementation of PCCS recommendations have rarely been published. Some data are available from the US: The most recent data we found were of Gupta et al. for 2014, who showed that opioid recommendations in an urban general hospital were implemented in about 60% of cases verbatim, in 20% with deviations and 20% not at all [20]. Earlier studies also report limited implementation, e.g. 73% for symptom management recommendations in an academic teaching hospital in the 90ies; [21] or 57% [22] and 84% [23] of all PCCS recommendations in veteran affair medical centers.

Research on the reasons for deviations and non-implementation is more common. Deviations often result from intentional decisions made by ward staff due to factors such as changes in or different assessment of the patient’s clinical condition, patient or family refusal [23, 24]. In these cases, deviations from a recommendation may or may not be a failure to provide adequate opioid therapy [25]. 

Another critical yet not well studied issue in clinical practice are unintentional deviations that are caused by errors in opioid therapy [25]. These errors can have significant consequences for patients, ranging from unnecessary symptom burden or side effects to life-threatening complications. Contributing factors for such errors include non-adherence to standards, distraction and workload [25], staff inexperience and insufficient supervision [26]. 

This online survey aims to explore the experiences of PCCS physicians regarding the implementation of their opioid recommendations in German hospitals: How often and what kind of deviations from their recommendations of WHO step III opioids do they observe? What reasons do they assume for these deviations? What measures are currently in place or should be implemented to improve challenges in collaborative opioid therapy?

## Methods

### Study design

 The study employed a closed online survey to collect data from PCCS in German hospitals using the ScoSciSurvey platform [27] The reporting adheres to the standards of the CHERRIES-Checklist [28]. 

### Sample

All PCCS in Germany listed as of October 2024 in the directory of the German Association for Palliative Medicine or included in a mailing list of the university palliative care working group were included. Survey responses were intended for completion by (senior) medical staff with at least one year of experience in the PCCS or collaboratively by team members. Each PCCS was allowed to submit only one completed survey.

### Survey process

 A database of all initially available PCCS email addresses was created; outdated addresses were updated through web searches, and non-existent facilities were excluded. Participation invitations were emailed on November 14, 2024, followed by a reminder on December 3, 2024. Personalized survey links, which allowed one entry only, were used to prevent duplicate submissions and allowed participants to pause and resume the survey. Links were not tracked to ensure anonymity. The survey concluded on December 31, 2024.

Participation invitations included detailed information about the study topic, process, time commitment, data usage and storage, and anonymity. Participants had the option to provide their email addresses to receive updates on survey results. Email addresses were stored separately. No incentives were offered.

### Questionnaire development and testing (detailed description in Additional File 1)

The initial questionnaire was developed by CR and EM based on relevant literature, local experiences, and discussions with representatives from the CCC WERA Palliative Medicine working group (Comprehensive Cancer Centers *W*uerzburg/*E*rlangen/*R*egensburg/*A*ugsburg). It was pre-tested with nine experienced palliative care physicians and one nurse specialist from six German and one Swiss site. The pilot testing included: (a) content validation, (b) assessing question clarity, relevance, and response scales through cognitive interviews with think-aloud and probing techniques and (c) technical functionality and usability.

### Questionnaire (full version included in Additional File 2)

The final version, included four sections spread over six online pages: ‘PCCS opioid recommendations’ (1 page, 4 items), ‘Implementation of recommendations’ (2 pages, 5 items), ‘Needs for improvement and measures for improvement’ (1 page, 3 items), ‘PCCS characteristics’ (1 page, 7 items) and ‘participant characteristics’ (1 page, 4 items). Four items included checks to flag unanswered questions, though participants could proceed without answering. Participants could revise their responses at any time.

### Data management and analysis

 Survey data were exported from ScoSciSurvey [27] in SPSS format and analyzed using IBM SPSS 29 [29]. Responses with more than 30% missing data on closed questions were excluded, no time limit for completion of the survey was set.

Three distinct PCCS groups were coded: (I) PCCS at university hospitals that provide opioid recommendations, (II) PCCS at general hospitals that provide opioid recommendations, and (III) PCCS at general hospitals that issue opioid prescriptions. None of the university hospital PCCS directly describes opioids. Differences between these three groups were examined using Chi-square test or H-Wallis H-test with post-hoc Dunn test, depending on the scale level. The significance threshold was set at 5% (two-tailed). Given the exploratory nature of the study, no Bonferroni correction was applied. Missing data were transparently reported.

We conducted a content analysis [30] of open-ended responses (detailed methods: Additional File 4). As the answers to open ended questions were short and items enquired on specific contents a simplified approach was taken to analyze them and results are fully and transparently reported: For each of the two relevant items a systematic inductive coding process was used. Responses were reviewed by one researcher (EM), key themes and recurring ideas were identified and all responses were then categorized into thematic groups based on the resulting category system. Responses were assigned to one or more categories as appropriate. The resulting category system and assignment of all the responses were reviewed by a second researcher (CR), discrepancies in categorization and coding were resolved through collaborative discussion.

## Results

### Participation and sample

Initially the database included 95 PCCS, for ten PCCS email addresses were non-functional; 85 email invitations were successfully sent (26 university hospital, 59 general hospital).

The survey link was accessed by 47 different PCCS (view rate: 55%), the questionnaire was started by 41 (of 47; participation rate: 87%). Two datasets were excluded due to over 30% missing data, leaving 39 (completion rate: 95%). Evaluable data were available for 46% (39 of 85; response rate) of all contacted PCCS (31% of general hospital PCCS (*n* = 18), and 81% of university hospital PCCS (*n* = 21)).

### Characteristics of participating PCCS (Table 1)

Most services operated within large hospitals with over 500 beds. All 21 university hospital PCCS and ten general hospital PCCS provided medication recommendations, eight general hospital PCCS prescribed medications. Notable differences were observed between university and general hospital PCCS, e.g. university hospital usually have higher capacities and Comprehensive Cancer Centers. In all cases, the survey was completed by senior physicians certified in palliative medicine.

**Table 1:** Characteristics of PCCS and respondents (Frequency *n*, percentage %)

	All participants	University Hospital	General Hospital			
		Recommendation (*n* = 21)	Recommendation (*n* = 10)	Prescription (*n* = 8)	Significance-test*	
Palliative care consultation service						
Hospital size, number of beds						
≤ 299	5	0	3	2	X^2^(2) = 39.00.; *p* <.001	
300 to 499	3	0	1	2		
500 to 699	7	0	5	2		
> 700 beds	24	21	1	2		
Comprehensive Cancer Center						
Yes	22	19	1	2	X^2^(2) = 20.78; *p* <.001	
No	16	2	8	6		
No answer	1	0	1	0		
Palliative care unit						
Yes	30	21	6	3	X^2^(2) = 14.92; *p* <.001	
No	9	0	4	5		
Existence of PCCS						
2 years or less	4	2	2	0		X^2^(2) = 1.96; *p* =.376
More than 2 years	35	1	8	8		
Respondent						
Gender						
Female	30	19	7	4	X^2^(2) = 5.71; *p* =.058	
Male	9	2	3	4		
Position						
Specialist doctor (lead)	31	16	9	6	X^2^(2) = 0.92; *p* =.632	
Specialist doctor (no lead)	8	5	1	2		
Specialist for…						
Internal medicine	21	12	7	2	X^2^(2) = 0.92; *p* =.632	
Anaesthesiology	16	7	3	6		
General medicine	2	2	0	0		
Additional qualification (more than one possible)						
Palliative medicine	39	21	10	8	--	
Emergency medicine	12	5	2	5	X^2^(2) = 4.80; *p* =.091	
Pain medicine	8	4	1	3	X^2^(2) = 2.12; *p* =.346	

*Chi-square test (nominal data) or Kruskal-Wallis H-test (ordinal data); significance level *p* <.05 (two-sided)

### Need for improvement (Fig. 1, detailed results in additional file 3 (1))

Twenty-three of the 39 PCCS reported a rather high or very high need for improvement in the implementation of strong opioid medication based on their recommendations. PCCS at university hospitals reported significantly higher levels of need for improvement (H (2)= 8.26, *p* =.016) compared to those PCCS in general hospitals that directly prescribe opioids (*z* = 11.76, *p* =.005).

**Fig. 1:**
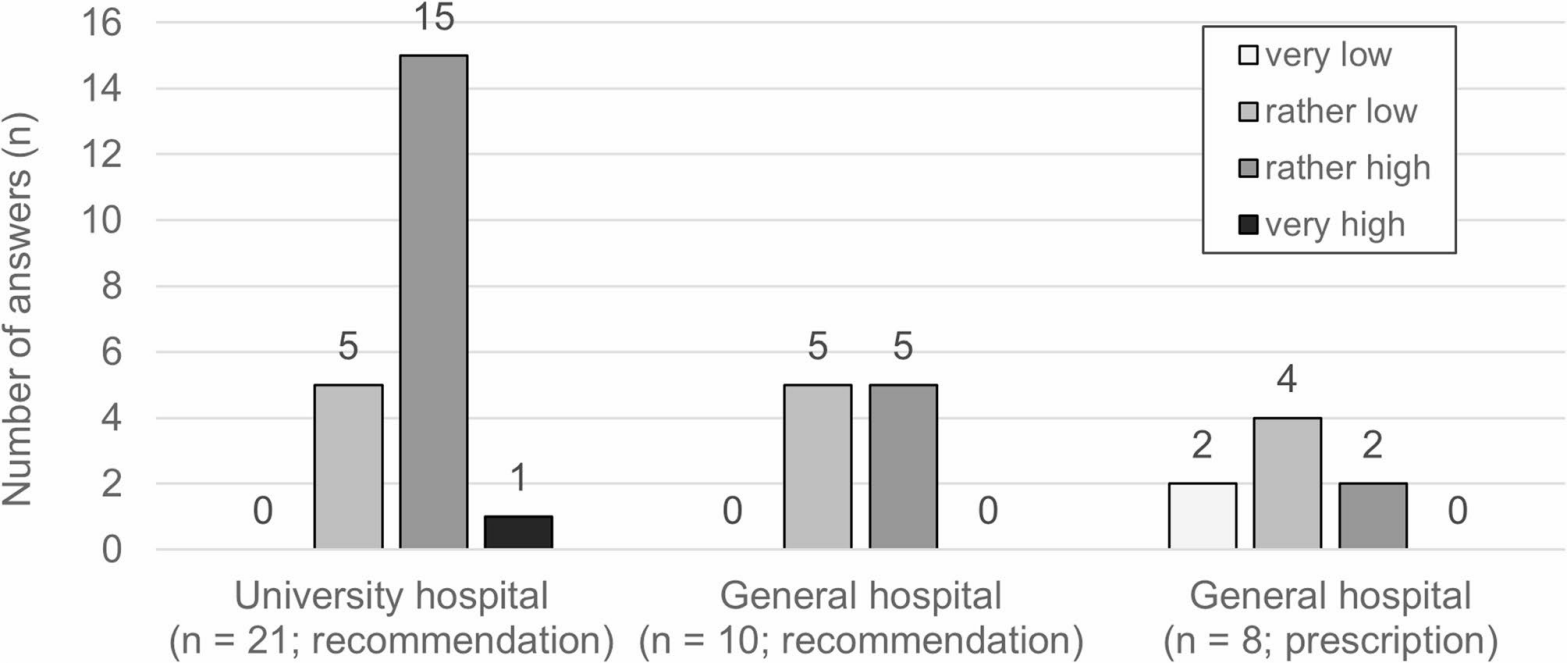
Need for improvement regarding implementation of medication with WHO step III opioids based on the PCCS recommendations depending on hospital type and recommendation vs. prescription of opioids

### Deviations from opioid recommendations (Fig. 2, additional file 3 (2))

 Overall, the most frequently reported deviations are “no co-medication for side-effects’, ‘no implementation at all’ and ‘lower dose’. PCCS that make recommendations reported deviations from opioid recommendations significantly more often than PCCS that prescribe opioids.

**Fig. 2:**
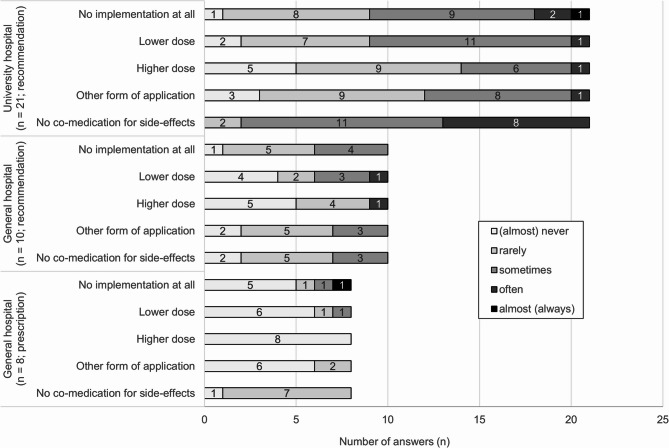
Deviations from opioid recommendations of the PCCS depending on hospital type and recommendation vs. prescription of opioids

### Reasons for the deviations (Fig. 3, additional file 3 (5))

Almost all PCCS (35/39) report “inexperience or reservations about opioid therapy among attending ward staff’ as a reason for deviations occurring ‘sometimes’ or ‘often’. This was followed by reasons indicating intentional non-implementation, such as “changed clinical status” and “differences in the assessment of the symptom burden between the PCCS and attending physicians.” No significant group differences were observed.

**Fig. 3:**
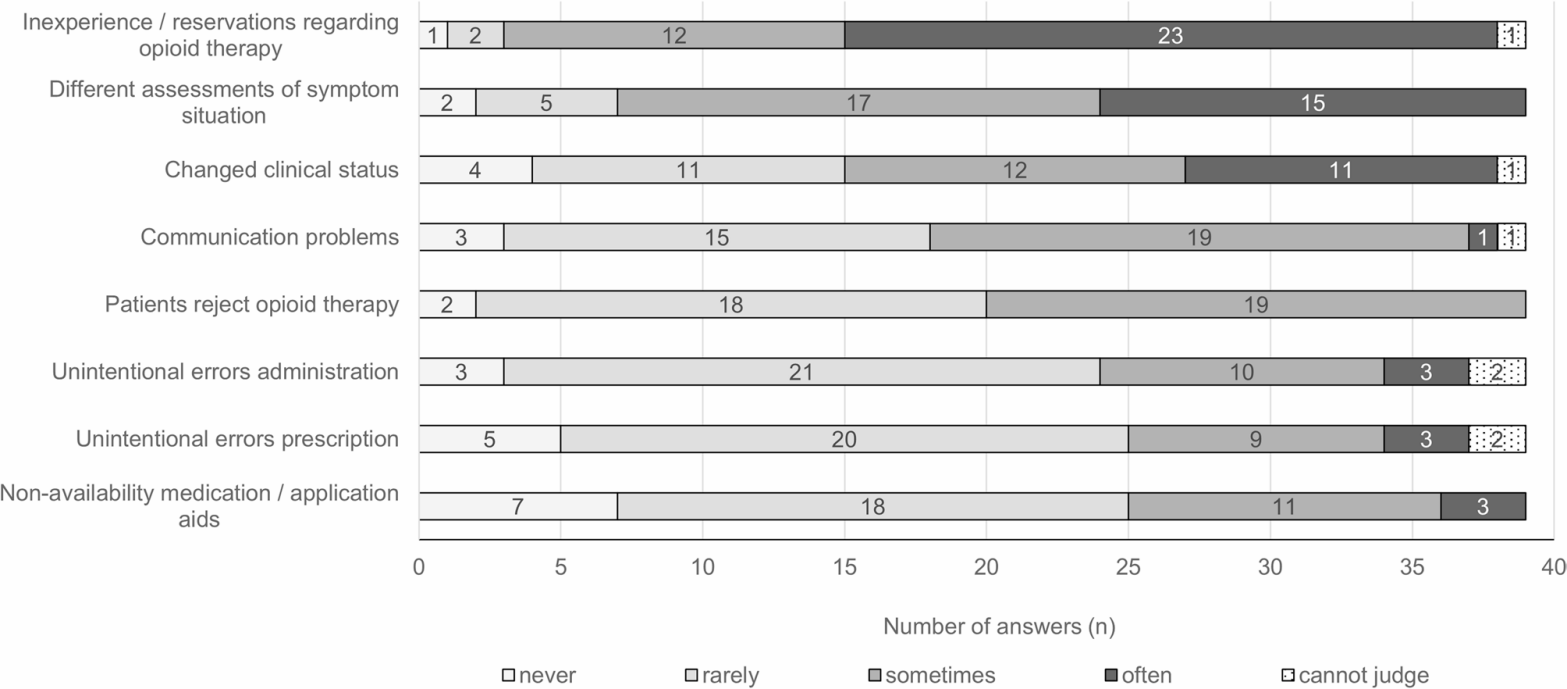
Reasons for the Deviations from opioid recommendation/prescription from the perspective of PCCS

### Errors in administration of opioids (Additional File 3 (3/4))

Fourteen out of 31 PCCS that provide recommendations reported observing unintentional dosing errors, eight drug application errors, and six drug mix-ups sometimes or often (5-point Likert-Scale: (almost) never/rare/sometimes/frequently/(almost) always)). In contrast, in the eight PCCS that prescribe opioids, all types of errors were rarely reported.

More than half of the PCCS (23/39) reported knowledge of cases of harm caused by opioid overdoses or situations of acute risk of harm within the last 12 months. University hospital PCCS reported such cases significantly more often than general hospital PCCS (H (2) = 9.91, *p* =.007); no differences were observed between general hospitals describing vs. recommending opioids.

### Inappropriate use of opioids (Additional File 3 (6))

Two-thirds of respondents reported witnessing opioid prescription “without indication or overdose at the end of life” (24 of 39 PCCS) ‘sometimes’, or ‘often’ (4-point Likert scale: never/rare/sometimes/often), 25 PCCS reported witnessing opioid use for “anxiety/restlessness,” and 21 for “sedation”. There were no significant differences between groups.

### Situations/topics of particular concern (Table 2, additional file 4)

Eighteen participants described in an open-ended question situations and topics that concern them regarding the opioid-therapy, many of them named more than one. The content analysis resulted in the two-level category system reported in Table 2.

**Table 2:** Categories of situations/topics of particular concern

Sub-category	Illustrating Quotes (translated from German):
Main Category: Areas for improvement	
Unsatisfactory implementation of PRN (pro re nata) medication	*“PRN medication administration is often poorly executed on general wards*,* especially before physical exertion or examinations.”*
Medication on discharge	*“[…] Not all colleagues or department heads are prepared to issue narcotic prescriptions.”*
Inappropriate opioid use	*“Cases of euthanasia with opioids carried out by attending physicians under pressure to free up hospital beds.”*
Unsatisfactory symptom assessment	*“[…] thorough symptom assessment by the ward staff […] enables quicker dose adjustments.”*
Errors in opioid rotation	*“Lack of knowledge about opioid equivalence doses on wards.”*
Errors in the use of perfusors	*“Better handling of opioid perfusors on general wards […].”*
Main category: Systemic and educational underlying factors	
Inexperience and lack of knowledge	*“Persistent myths among professionals*,* family members*,* and patients that opioids accelerate or induce death.”*
Resistance to training/advice	*“Department heads show little openness to existing training opportunities.”* *“A significant concern is colleagues who are very resistant to advice in their handling of opioids*,* which poses a danger […] for patients.”*
Heterogeneous implementation across departments	*“Departments visited less frequently (e.g.*,* cardiology*,* thoracic surgery*,* intensive care units) are the most hesitant to implement recommendations.”*
Limited opioid availability	*“Restrictions in opioid selection due to stock limitations in the hospital pharmacy.”*
Staff turnover	*“[…] individualized concepts are challenging to implement due to frequent personnel turnover in both nursing and medical staff.”*
Limitations of clinical information system	*“System limitations in clinical information systems (CIS)*, e.g.,* perfusors can only be prescribed in ml/h rather than mg/h*,* among other issues.”*

### Measures to improve implementation and opioid therapy (Additional file 3 (7)/4)

 Many PCCS reported “monitoring the implementation of opioid therapy,” (*n* = 28 answered “yes”), “monitoring symptom/side-effects,” (*n* = 30) and “open and uncomplicated communication” (*n* = 25) to improve opioid therapy implementation. Less common are measures such as “positive error culture” (*n* = 15), “training” of attending ward staff (*n* = 13), “hospital-wide standardization of opioid therapy” (*n* = 8) and “joint ward rounds” (*n* = 6).

Twenty-one participants answered the open-ended question, many suggesting multiple measures. Table 3 summarized the results of the content analysis (Additional File 4). Many answers align with themes identified in closed-ended questions. Newly added suggestions include more staff and time ‘resources’ and ‘internal support processes’ in attending wards.

**Table 3:** Results of content analysis of open-ended questions on measures to improve adherence to opioid recommendations

Sub-category	Illustrating quotes (translated from German)
Main category: Measures in attending wards	
Education and training	*“Ward-specific training*,* both in practical application and in addressing fears.”*
Internal support processes	*“Greater support for junior physicians from experienced specialists.”* *“Palliative care nurses on all wards to act as multipliers.”*
Main category: Measures in PCCS	
Independent prescribing authority for PCCS.	*“Granting the palliative care team autonomy to initiate opioid therapy independently (“opioid authority”).”*
Educational role as part of self-image	*“The palliative care team also serves an educational role for staff.”*
Main category: Measures in collaboration of attending wards and PCCS	
Monitoring by palliative care services.	*“The PCCS sees all involved patients […] daily*,* allowing discrepancies and errors to be identified and communicated promptly.”*
Integrated/interdisciplinary care	*“Education/further training within an integrated-consultative model enhances security for patients.”*
Uniform protocols/standard operating procedures	*“Recommendations for a standardized pain management concept for palliative patients*, e.g.,* in a card format.”*
Positive error culture	*“Open communication and a positive error culture throughout the hospital.”*
Timely integration of palliative care	*“Changing the perception of “palliative care” as purely end-of-life care*,* especially among colleagues*,* would help many patients much earlier.”*
Main category: hospital-wide/system	
Staffing and Resources as basic requirement for improved care.	*“Increased personnel and time resources.”* *“Most importantly: public education and funding for speaking medicine […].”*

## Discussion

### Need for improvement

Overall, most PCCS reported a high or very high need for improvement regarding the collaboration between PCCS and attending ward staff in opioid therapy, with sometimes high rates of non-implementation of or deviations from PCCS opioid recommendations. Our findings align with the U.S. data that also reflect varying and sometimes rather low implementation rates between hospitals [20–22]. 

The results of our survey indicate both too cautious and too incautious opioid usage. Frequently reported deviations from PCCS opioid recommendations are “no implementation at all” and “lower dose”. These often reflect intentional deviations and cautious use of opioids [7–11]. However, non-implementation may also be due to limited staff resources leading to recommendations being overlooked or implementation being forgotten [26, 31]. Further relevant topics are unintended medication errors in drug prescription/administration and inappropriate use e.g. without medical indication at the end of life.

Survey participants highlighted specific situations needing improvement, such as:

- symptom assessment for the adjustment and control of opioid therapy,
- the prescription and use of PRN (pro re nata; on-demand) medication to manage unexpected and expected symptom exacerbations e.g. during procedures,
- continuing prescription and administration of opioid therapy at discharge,
- inappropriate opioid management e.g. during the dying phase.

### Influencing factors

The type of hospital and the question who prescribes the opioids – PCCS vs. attending physician after PCCS recommendation — contribute significantly to the variations between hospitals.

The university/high-capacity hospitals reported significantly more problems. Several factors are likely to contribute to that result: On average university/high-capacity hospitals handle more severe cases and they serve as training centers. This implies a rather high proportion of inexperienced staff, frequent staff rotations and enhanced need for supervision, which is hard to provide given the shortage of skilled healthcare professionals and can negatively influence care processes and patient outcomes [32, 33]. Furthermore, the larger the hospitals, the less likely are close relationships among staff from different departments, which can lead to more challenging collaborations.

A higher need for improvement is reported in those hospitals where attending physicians are required to prescribe the opioids recommended by the PCCS. In this case the attending physician bears the legal and medical responsibility. In contrast, if the PCCS directly prescribes opioids, the legal and medical responsibility falls on the PCCS physician, and the medication is expected to be administered unless there are serious concerns. However, opioid prescribing by the PCCS is difficult to implement in large hospitals: long distances and the high number of patients make regular monitoring by the PCCS challenging.

### Improvement measures

Therapy with potent opioids is complex due to dosage adjustments, side effect management, and patient-specific factors to take into account for choice of drug and dose. While attending physicians are not required to handle complex cases independently, they should be confident to provide appropriate therapy in collaboration with the PCCS team.

Insufficient experience and knowledge are reported to be key reasons for deviations from opioid recommendations in our survey. This PCCS perspective is confirmed from the ‘other side’, as in numerous studies the attending physicians themselves report lacking knowledge and confidence, even in intensive care and oncology [34–37]. Despite palliative care, including basic knowledge regarding opioids, being part of German medical training since 2009, a theory-practice gap persists [38], partly because many supervising physicians remain untrained. The most frequently suggested measures for improvement are the various formal and informal educational approaches. Artificial intelligence (AI) might contribute the opportunity of more effective and targeted training in times of shortage of qualified staff [39]. 

Various, often hospital-, sometimes even department-specific, issues in the collaboration between PCCS and attending wards contribute to the challenges in opioid therapy. Proposed measures include standardized opioid protocols, fostering a positive error culture [40], and joint ward rounds to enhance therapy, support knowledge transfer, and strengthen relationships between teams [41]. 

### Strengths and limitations

The use of an anonymous survey allowed us to explore a sensitive and controversial topic. Some aspects, such as witnessing intentional or unintentional opioid overdoses, touch on legal issues and are typically kept quiet in everyday practice [31]. 

A strength of our study is the systematic development and pilot testing of the questionnaire, which ensured content validity, clarity, and usability. The relatively high participation and completion rates, along with engaged responses to open-ended questions, indicate the topic’s relevance and the questionnaire’s clarity. Additionally, all participants who completed the survey as representatives of their PCCS were experienced, well-trained and thereby likely to be able to evaluate the topic of the survey.

The overall response rate of 46% is moderate. While it was very high for university hospitals (81%), it was only 31% for general hospitals, specifically limiting the generalizability of findings for PCCS based there. There are indications that invitation emails to some general hospital PCCS were classified as spam. Additionally, the authors had prior contact with all university hospital PCCS, reducing the likelihood of emails being classified as spam. Furthermore, although the PCCS directory of the German Association for Palliative Medicine is the main registry of palliative services, registration is voluntary, so our survey may also not have reached all relevant providers.

The results of this study reflect collaboration challenges regarding opioid therapy in Germany and cannot be generalized to other countries or settings, However, similar issues may exist in other resource-rich health systems with established in-hospital consultation services.

Another possible limitation of the results of this survey is response bias, as participants’ answers may not fully reflect reality but be biased through reactions towards the questionnaire content. Further research, such as chart reviews, is needed to confirm the results.

A key limitation of this study is the lack of input from attending ward staff. Only PCCS were surveyed to report their experiences and practices. Further studies should add that perspective to allow for an in-depth exploration of challenges in collaboration and need for improvement in symptom management. For example, studies show that inadequate, unclear or delayed recommendations on the side of the PCCS contribute to problems in opioid therapy [42]. 

## Conclusion

Physicians in PCCS report significant challenges in implementing their opioid therapy recommendations, with overly cautious prescribing being the most commonly observed issue. This is particularly evident in university and high-capacity hospitals, where often the patients with the most complex needs are treated and collaboration between PCCS and attending wards tends to be more difficult.

Targeted education on opioid therapy for attending ward staff is the most frequently suggested improvement measure. Additionally, proposed strategies focus on enhancing collaboration — both within care teams and between PCCS and attending wards — such as through improved monitoring of opioid therapy by PCCS and fostering a positive error culture. To address these issues effectively, it is essential to consider and integrate the perspectives of attending ward teams.

## Supplementary Information

Additional file 1: Questionnaire Development. Description of methods and results of the development and pre-testing of the survey items.

Additional file 2: Questionnaire. Full questionnaire including all items.

Additional file 3: Statistics. Detailed statistics of all results, mostly tables, in some cases figures.

Additional file 4: Content analysis of answers to open ended questions. Detailed description of methods and results of content analysis of open-ended answers; including original data.

## Data Availability

The datasets analyzed during the current study are available from the corresponding author on reasonable request.

## Abbreviations

PCCS: palliative care consultation services
