# Supplementary material for: Challenges in opioid therapy implementation: national survey of palliative care consultation services
Source: BMC Palliat Care. 2025 Oct 20;24:262. doi: 10.1186/s12904-025-01921-0 (PMC12539157; doi:10.1186/s12904-025-01921-0)
Supplement: Supplementary file 1 — Additional file 1: Questionnaire Development. Description of methods and results of the development and pre-testing of the survey items. [file 12904_2025_1921_MOESM1_ESM.pdf]

Additional File 1 – Questionnaire Development and Pilot Testing

This document outlines the iterative development process, rigorous testing, and refinements made to ensure the reliability and relevance of the questionnaire.

Methods

Initial Version of the Questionnaire: The first draft of the questionnaire was developed by CR and EM based on relevant literature, local experiences, and initial project discussions with representatives from the Palliative Medicine Working Group of the CCC WERA network (Würzburg/Erlangen/Regensburg/Augsburg).

Pilot Testing: The questionnaire was iteratively refined through testing with physicians and nurses from multiple locations, all of whom had several years of experience in palliative care services (PCS). The pilot testing included:

> (a) Content Validation: Open questions were used to explore participants' perspectives on the topics of "opioid recommendations," "implementation of opioid recommendations by attending wards," and "measures to improve implementation and treatment safety".
>
> (b) Evaluation of Clarity and Feasibility: Cognitive interviews employing think-aloud protocols and probing were conducted to assess the clarity, answerability, and alignment of questions and response scales.

Pilot testing sessions were conducted either in person or via online meetings. The content areas tested included "opioid recommendations," "implementation of opioid recommendations by attending wards," and "measures to improve implementation and treatment safety." Structural questions about PCS and sociodemographic or professional details of the respondents were not included in the pilot testing. After each testing round, improvements were implemented and tested in subsequent iterations. Pilot testing concluded when no further issues were identified regarding clarity or answerability and when no new thematic aspects emerged from the content validation.

Online Pretest: The functionality of the online implementation was validated in a pretest. This included internal team reviews followed by external testing by physicians.

Results

Sample: The questionnaire was tested with nine physicians and one nurse, all with extensive experience in PCS. Participants worked in palliative care services at six German and one Swiss hospital. The sample included six women and four men, seven of whom held leadership roles in their PCS. All participating physicians were board-certified with additional qualifications in palliative medicine.

Sessions: Three testing sessions were conducted in person, and seven were held online. Nine sessions used Word-based tabular formats resembling the online implementation, while one utilized the final online version on ScoSciSurvey.com.

Tab. S2.1: Examples of Revisions Based on Pilot Testing:

Objective of the Change	**Examples**
Addition of new content to existing items	*Deviations in implementation:* opioid administration without co-medication for side-effect prophylaxis.<br>*Measures for improvement:* Promotion of active communication and joint ward rounds
Linguistic changes for clarity	Added the term "WHO Step 3" in parentheses to clarify the meaning of "potent opioids<br>Reworded "non-guideline-conforming or inappropriate use" to "non-standard use (‘off-label use’)”
Inclusion of a time frame	Changed "currently" to "in the past 12 months" for questions about improvement measures.
Deletion of items	Removed multi-part questions on detailed communication between PCS and attending wards in favor of a single, more comprehensive item. |

Newly Introduced Topic: The topic of “inappropriate use of strong opioids in attending wards” was included in the survey due to its frequent mentions and high relevance to interviewees during pre-testing. This topic covers both the use of opioids for indications where guidelines recommend alternative medications (e.g., benzodiazepines for anxiety and restlessness) and the use of opioids without any clear indication. It was included because of its importance for ensuring safe opioid therapy in generalist palliative care, even though such use is not recommended by PCS in these cases.

Topics discussed during questionnaire development but not included: Some topics were considered insufficiently relevant and were therefore excluded: Co-medication with non-opioid analgesics or other agents (e.g., antidepressants), non-standard opioid use for purposes such as physician-assisted suicide or active euthanasia and measures to improve treatment safety or implement opioid recommendations that are beyond the influence of PCS (e.g. solving internal communication issues on the wards or problems with the computer software).

Online Pretest: During the online pretest, a technical function resulted in open-ended questions being classified as "mandatory," prompting participants to complete these before progressing. Due to the lack of technical options to enforce mandatory Likert-scale items while keeping open-ended questions optional, all items that included open-ended questions were reclassified as "optional."
