# Supplementary material for: Challenges in opioid therapy implementation: national survey of palliative care consultation services
Source: BMC Palliat Care. 2025 Oct 20;24:262. doi: 10.1186/s12904-025-01921-0 (PMC12539157; doi:10.1186/s12904-025-01921-0)
Supplement: Supplementary file 2 — Additional file 2: Questionnaire. Full questionnaire including all items. [file 12904_2025_1921_MOESM2_ESM.pdf]

Additional File 2 – Questionnaire (translated from German)

Recommendations on strong opioids

**1.**	**Please estimate for what proportion of patients receiving palliative care does your palliative care service recommend strong opioids (WHO level 3)?**				
☐	☐	☐	☐	☐	O
0 - 20 %	21 - 40 %	41 - 60 %	61 - 80 %	81 - 100 %	I cannot judge

**2.**	**For which indications do you recommend treatment with strong opioids (WHO level 3)?** Several statements possible.
☐	Tumour pain
☐	Non-tumour pain
☐	Respiratory distress in patients with tumour disease
☐	Shortness of breath in patients with pneumological diseases (e.g. COPD)
☐	Shortness of breath in patients with cardiological diseases (e.g. heart failure)
☐	Other:

3. How often does your palliative care service use the following medications in the recommendations?

	never	rare	sometimes	frequently	I cannot judge
Morphine	☐	☐	☐	☐	O
Hydromorphone	☐	☐	☐	☐	O
Fentanyl	☐	☐	☐	☐	O
Oxycodone	☐	☐	☐	☐	O
Buprenorphine	☐	☐	☐	☐	O
L-polamidone	☐	☐	☐	☐	O
Piritramide	☐	☐	☐	☐	O
Pethidine	☐	☐	☐	☐	O
Other:____________________	☐	☐	☐	☐	O

© Interdisciplinary Center for Palliative Medicine, Department of Radiation Oncology, CCC Mainfranken University Hospital Wuerzburg,

4. How often does your palliative care service use the different forms of administration in the opioid recommendations?	never	rare	sometimes	frequently	I cannot judge
orally retarded	☐	☐	☐	☐	O
orally acute	☐	☐	☐	☐	O
subcutaneous	☐	☐	☐	☐	O
transdermal	☐	☐	☐	☐	O
intravenous	☐	☐	☐	☐	O
buccal	☐	☐	☐	☐	O
nasal	☐	☐	☐	☐	O

Implementation of the recommendations

5. How often do the following deviations from the palliative care service recommendations occur during implementation?

**The recommended highly effective opioids are...**	(almost) never	rare	sometimes	frequently	(almost) always	I cannot judge
not implemented at all	☐	☐	☐	☐	☐	O
applied in a lower dosage	☐	☐	☐	☐	☐	O
applied in higher dosage	☐	☐	☐	☐	☐	O
Other application form implemented	☐	☐	☐	☐	☐	O
implemented without recommended co-medication to control side effects (e.g. laxatives)	☐	☐	☐	☐	☐	O
Other: _________________	☐	☐	☐	☐	☐	O

6. How often do errors occur when administering strong opioids based on palliative care recommendations?

	(almost) never	rare	sometimes	frequently	(almost) always	I cannot judge
Wrong drug	☐	☐	☐	☐	☐	O
Dosing errors	☐	☐	☐	☐	☐	O
Application error	☐	☐	☐	☐	☐	O
Other: _________________	☐	☐	☐	☐	☐	O

© Interdisciplinary Center for Palliative Medicine, Department of Radiation Oncology, CCC Mainfranken University Hospital Wuerzburg,

7. **How many cases in the past 12 months are you aware of where patients were harmed or endangered by an opioid overdose?**					
☐	☐	☐	☐	☐	O
Not a single	1 - 2 cases	3 - 5 cases	6 - 10 cases	> 10 cases	I cannot judge

8. What reasons do you see for deviations in the implementation of the recommendations on strong opioids on the wards?

	**never**	**rare**	**sometimes**	**frequently**	**I cannot judge**
Changed clinical status of the patient	☐	☐	☐	☐	O
Different assessments of the symptom situation by the attending ward and palliative care service	☐	☐	☐	☐	O
Inexperience / reservations regarding opioid therapy	☐	☐	☐	☐	O
Unintentional errors in the prescription in the curve (e.g. due to typing errors, incorrect entry)	☐	☐	☐	☐	O
Unintentional errors during administration (e.g. incorrect dose, application)	☐	☐	☐	☐	O
Problems in communication between palliative care service and attending ward	☐	☐	☐	☐	O
Patients / relatives reject opioid therapy	☐	☐	☐	☐	O
Non-availability of medication and / or application aids	☐	☐	☐	☐	O
Other:___________________	☐	☐	☐	☐	O

9. Is inappropriate use of strong opioids by wards an issue facing your palliative care service?

**We learn of cases of opioid therapy ....**	never	rare	sometimes	frequently	I cannot judge
for sedation	☐	☐	☐	☐	O
for anxiety / restlessness	☐	☐	☐	☐	O
overdose or prescription without indication in the dying phase	☐	☐	☐	☐	O
Other:___________________	☐	☐	☐	☐	O

© Interdisciplinary Center for Palliative Medicine, Department of Radiation Oncology, CCC Mainfranken University Hospital Wuerzburg,

Need for improvement and improvement measures

**10. In your opinion, how great is the need for improvement with regard to the implementation of medication with strong opioids based on the palliative care service recommendations?**				
very low	rather low	rather large	very large	I cannot judge
☐	☐	☐	☐	O

Below you will find possible measures through which palliative care services can possibly promote the implementation of the recommendations and treatment safety in opioid therapy.

**11. Are these measures currently being implemented in your clinic (last 12 months)? If not, would you find them useful?**				
	**yes**	**partly**	**no**	
			would make sense	would not make sense
Training of users on the wards by the palliative care service	☐	☐	☐	☐
Monitoring of the implementation of opioid recommendations by the palliative care service	☐	☐	☐	☐
Monitoring of symptom control and side effects of opioid therapy by the palliative care service	☐	☐	☐	☐
Clinic-wide standardisation of opioid therapy (e.g. uniform concentrations for intravenous / subcutaneous administration)	☐	☐	☐	☐
Open and uncomplicated communication for questions and problems (e.g. "just call the palliative care service")	☐	☐	☐	☐
Positive error culture (e.g. joint review of errors)	☐	☐	☐	☐
Joint ward rounds	☐	☐	☐	☐
Other:___________________	☐	☐	☐	☐

12. Are there any important aspects of the topic that have not yet been mentioned? Which situations / topics relating to opioid therapy are of particular concern to you?

13. If you could freely wish for something to improve these situations/issues: What would you wish for?

© Interdisciplinary Center for Palliative Medicine, Department of Radiation Oncology, CCC Mainfranken University Hospital Wuerzburg,

Your palliative care service

14. Our palliative care service makes	
☐	The recommendations on opioids, the orders are the responsibility of the ward doctors
☐	Directly the disposition of opioids

15. Our care service is	
☐	An internal structure at a hospital
☐	External service provider (e.g. SAPV) and performs consultations at one or more clinics

16. Our palliative care service has existed since	
☐	2 years or less
☐	more than 2 years
☐	not known

17. Our PD is at a hospital on the level of...	
☐	basic care (up to 299 beds)
☐	standard care (300 to 499 beds)
☐	centralised care (500 to 699 beds)
☐	maximum care (700 to over 1000 beds)

18. Our palliative care service is	
☐	University Hospital
☐	General hospital (teaching)
☐	General hospital (not teaching)

19. Our palliative care service is based at a clinic with a Comprehensive Cancer Centre	
☐	Yes
☐	No

20. There is a palliative care ward in our hospital	
☐	Yes
☐	No

© Interdisciplinary Center for Palliative Medicine, Department of Radiation Oncology, CCC Mainfranken University Hospital Wuerzburg,

Your person

If you answered with more than one person, please refer to the person with the most experience with the topic of the survey:

**21. Your gender**
☐ Female
☐ Male
☐ Diverse

**22. Your profession:**
☐ Specialist doctor in a leading position
☐ Specialist doctor without management position
☐ Physician in training
☐ Other__________________

**23. Specialist for**
☐ Anaesthesiology
☐ General medicine
☐ Internal medicine
☐ Neurology
☐ Surgery
☐ Other:_____________________

**24. Additional qualification**
☐ Palliative medicine
☐ Emergency medicine
☐ Pain therapy
☐ Other:_____________________

**25. Is there anything else you want to tell us?**

© Interdisciplinary Center for Palliative Medicine, Department of Radiation Oncology, CCC Mainfranken University Hospital Wuerzburg,
