# Supplementary material for: Challenges in opioid therapy implementation: national survey of palliative care consultation services
Source: BMC Palliat Care. 2025 Oct 20;24:262. doi: 10.1186/s12904-025-01921-0 (PMC12539157; doi:10.1186/s12904-025-01921-0)
Supplement: Supplementary file 3 — Additional file 3: Statistics. Detailed statistics of all results, mostly tables, in some cases figures. [file 12904_2025_1921_MOESM3_ESM.pdf]

Additional File 3: Statistics

(1) Need for improvement with regard to implementation of medication with strong opioids

Tab. S1.1: Descriptive data regarding need for improvement

	In your opinion, how great is the need for improvement with regard to the implementation of medication with strong opioids based on the palliative care service recommendations?			
	**very low**	**rather low**	**rather high**	**very high**
**Total Sample (n = 39)**	2	14	22	1
**University Hospital (n = 21; recommendation)**	0	5	15	1
**General Hospital (n = 10; recommendation)**	0	5	5	0
**General Hospital (n = 8; prescription)**	2	4	2	0

Tab. S1.2: Kruskal-Wallis-H-Test to check for group differences in need for improvement

	Need for improvement	
**Kruskal-Wallis H-Test (df = 2)**	**H**	**p**
	8,259	0.016*
**Post-Hoc-Dunn-Test**	**z**	**p**
General hospital PCS (prescription) vs. general hospital PCS (recommendation)	6.500	0.172
General hospital PCS (prescription) vs. university hospital PCS (recommendation)	11.762	0.005‘
General hospital PCS (recommendation) vs. university hospital PCS (recommendation)	5.262	0.172

* significant on a level of p < .05 (two-sided), ** significant on a level of p < .01 (two-sided),

(2) Deviations between opioid recommendations of palliative care consultation team and implementation

Tab. S2.1: Descriptive data regarding deviations from opioid recommendations for the full sample (n=39)

	How often do the following deviations from the palliative care service recommendations occur during implementation?				
	**No implement-tation at all**	**Lower dose**	**Higher dose**	**Other form of application**	**No co-medication for side-effects**
(almost) never	7	12	18	11	3
rarely	14	10	13	16	14
sometimes	14	15	6	11	14
often	2	2	2	1	8
almost (always)	2	0	0	0	0

Tab. S2.2: Kruskal-Wallis-H-Test to check for group differences in report of deviations

	**Deviation**									
	**No implement-tation at all**		**Lower dose**		**Higher dose**		**Other form of application**		**No co-medication for side-effects**	
**Kruskal-Wallis H-Test (df = 2)**	**H**	**p**	**H**	**p**	**H**	**p**	**H**	**p**	**H**	**p**
	5.603	0.061	8.761	0.013*	12.135	0.002**	10.316	0.006**	19.374	0.000**
**Post-Hoc-Dunn-Test**	z	p	z	p	z	p	z	p	z	p
General hospital PCS (prescription) vs. general hospital PCS (recommendation)	6.363	0.215	8.138	0.112	9.100	0.070	11,475	,024*	6.438	0.208
General hospital PCS (prescription) vs. university hospital PCS(recommendation)	10.551	0.019*	13.188	0.003**	15.167	0.001**	14,268	,001**	18.176	0.000**
General hospital PCS (recommendation) vs. university hospital PCS (recommendation)	4.188	0.314	5.050	0.224	6.067	0.136	2,793	,498	11.738	0.005**

* significant on a level of p < .05 (two-sided), ** significant on a level of p < .01 (two-sided),

(3) Unintentional errors when administering the opioid

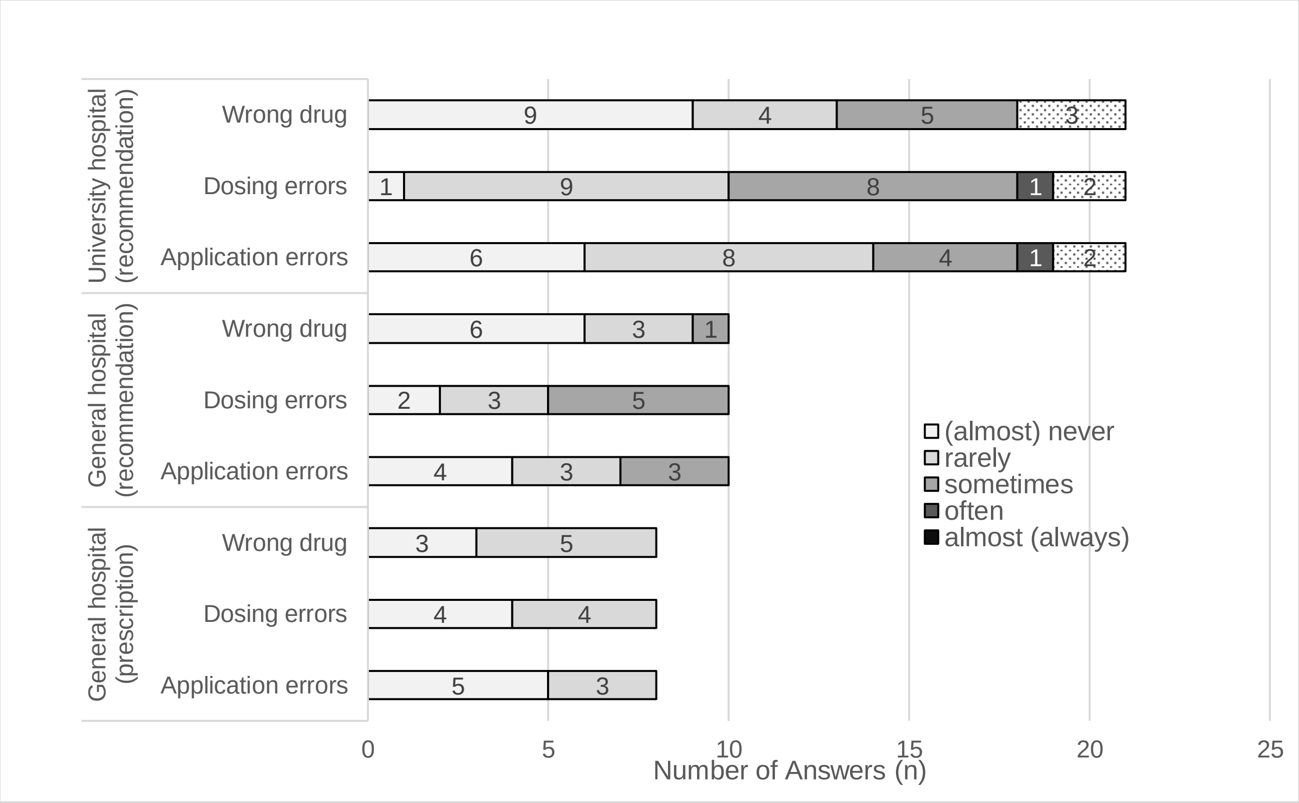

Fig. S3.1: Group-specific descriptive statistics of perceptions of administration errors of strong opioids in attending wards

Tab. S3.1: Kruskal-Wallis-H-Test to check for group differences in errors administering the drug

	Unintentional errors when administering the opioid					
	Wrong drug		Errors in dosage		Errors in application	
**Kruskal-Wallis H-Test (df = 2)**	**H**	**p**	**H**	**p**	**H**	**p**
	0.683	0.711	8.753	0.013*	3.157	0.206
**Post-Hoc-Dunn-Test**	**z**	**p**	**z**	**p**	**z**	**p**
General hospital PCS (prescription) vs. general hospital PCS (recommendation)			10.700	0.025*		
General hospital PCS (prescription) vs. university hospital PCS(recommendation)			12.382	0.004*		
General hospital PCS (recommendation) vs. university hospital PCS (recommendation)			1.682	0.670		

* significant on a level of p < .05 (two-sided), ** significant on a level of p < .01 (two-sided),

(4) Cases of opioid overdoses

Tab. S4.1: Descriptive data regarding awareness of cases of opioid overdose

	How many cases in the past 12 months are you aware of where patients were harmed or endangered by an opioid overdose?				
	no case	1-2 cases	3-5 cases	6-10 cases	cannot say
**Total sample (n=39)**	13	17	5	1	3
**University hospital (n = 21; recommendation)**	2	11	4	1	3
**General hospital (n = 10; recommendation)**	6	3	1	0	0
**General hospital (n = 8; prescription)**	5	3	0	0	0

Tab. S4.2: Kruskal-Wallis-H-Test to check for group differences in need for improvement

	Need for improvement	
**Kruskal-Wallis H-Test (df = 2)**	**H**	**p**
	9.906	.007**
**Post-Hoc-Dunn-Test**	**z**	**p**
General hospital PCS (prescription) vs. general hospital PCS (recommendation)	1.475	.748
General hospital PCS (prescription) vs. university hospital PCS (recommendation)	10.931	.008**
General hospital PCS (recommendation) vs. university hospital PCS (recommendation)	9.456	.013*

* significant on a level of p < .05 (two-sided), ** significant on a level of p < .01 (two-sided),

(5) Reasons for the deviations

Tab. S5.1: Group-specific descriptive statistics of assumed reasons for the deviations

		What reasons do you see for deviations in the implementation of the recommendations on strong opioids on the wards?				
		**never**	**rarely**	**some-times**	**often**	**cannot judge**
**Total sample (n=39)**	Non-availability medication / application aids	7	18	11	3	0
	Patients reject opioid therapy	2	18	19	0	0
	communication problems	3	15	19	1	1
	Unintentional errors administration	3	21	10	3	2
	Unintentional errors prescription	5	20	9	3	2
	Inexperience / reservations regarding opioid therapy	1	2	12	23	1
	Different assessments of the symptom situation	2	8	20	9	0
	Changed clinical status	4	11	12	11	1
**University hospital (n = 21; recom-mendation)**	Non-availability medication / application aids	4	6	8	3	0
	Patients reject opioid therapy	1	9	11	0	0
	communication problems	1	9	9	1	1
	Unintentional errors administration	1	10	6	3	1
	Unintentional errors prescription	4	8	5	3	1
	Inexperience / reservations regarding opioid therapy	0	1	8	11	1
	Different assessments of the symptom situation	0	4	10	7	0
	Changed clinical status	2	5	6	7	1
**General hospital (n = 10; recom-mendation)**	Non-availability medication / application aids	0	8	2	0	0
	Patients reject opioid therapy	1	6	3	0	0
	communication problems	1	2	7	0	0
	Unintentional errors administration	2	4	3	0	1
	Unintentional errors prescription	1	5	3	0	1
	Inexperience / reservations regarding opioid therapy	0	0	2	8	0
	Different assessments of the symptom situation	1	3	6	0	0
	Changed clinical status	2	3	3	2	0
**General hospital (n = 8; prescription)**	Non-availability medication / application aids	3	4	1	0	0
	Patients reject opioid therapy	0	3	5	0	0
	communication problems	1	4	3	0	0
	Unintentional errors administration	0	7	1	0	0
	Unintentional errors prescription	0	7	1	0	0
	Inexperience / reservations regarding opioid therapy	1	1	2	4	0
	Different assessments of the symptom situation	1	1	4	2	0
	Changed clinical status	0	3	3	2	0

Tab. S5.2: Kruskal-Wallis-H-Test to check for group differences in assumed reasons for the deviations

	Assumed reasons for the deviations							
**Kruskal-Wallis H-Test (df = 2)**	**Changed clinical status**	**Different assess-ments of symptom situation**	**Inexperience/ reservations Opioid therapy**	**Unintentional errors in prescription**	**Unintentional errors in administration**	**Communication problems (palliative care service – attending wards)**	**Patient / relatives refuse opioid therapy**	**Non-availability of medication / aids for administration**
H	1.064	2.380	2.729	0.337	2.589	1.464	2.365	2.365
p	0.587	0.304	0.256	0.845	0.274	0.481	0.307	0.307

* significant on a level of p < .05 (two-sided), ** significant on a level of p < .01 (two-sided),

(6) Inappropriate use of strong opioids in attending wards

Tab. S6.1: Group-specific descriptive statistics of perceptions of opioid inappropriate use of strong opioids in attending wards

		Is inappropriate use of strong opioids by wards an issue facing your palliative care service?			
		**never**	**rarely**	**sometimes**	**often**
**General hospital (n = 8; prescription)**	Sedation	1	2	5	0
	Anxiety / restlessness	0	3	5	0
	Prescription without indication / overdose at the end of life	2	3	3	0
**General hospital (n = 10; recommendation)**	Sedation	1	3	6	0
	Anxiety / restlessness	2	1	6	1
	Prescription without indication / overdose at the end of life	2	3	3	2
**University hospital (n = 21; recommendation)**	Sedation	1	10	6	4
	Anxiety / restlessness	3	5	6	7
	Prescription without indication / overdose at the end of life	3	2	11	5

Tab. S6.2: Kruskal-Wallis-H-Test to check for group differences in report of off-label use

	Unintentional errors when administering the opioid					
	**Sedation**		**Anxiety / restlessness**		**Prescription without indication / overdose at the end of life**	
**Kruskal-Wallis H-Test (df = 2)**	**H**	**p**	**H**	**p**	**H**	**p**
	0.016	0.992	0.603	0.740	3.795	0.150

* significant on a level of p < .05 (two-sided), ** significant on a level of p < .01 (two-sided),

(7) Measures to improve implementation

Tab. S7.1: descriptive statistics of use of measures to improve implementation

		Yes	Partly	No	
				would make sense	would not make sense
Total Sample (n=39)	Joint ward rounds	6	14	16	3
	Clinic-wide standardization of opioid therapy	8	14	10	7
	Training users	13	16	9	1
	Positive error culture	15	12	12	0
	Open / uncomplicated communication	25	10	3	1
	Monitoring implementation	28	4	4	3
	Monitoring symptom control & side effects	30	6	2	1

Tab. S7.2: Kruskal-Wallis-H-Test to check for group differences in use of measures to improve implementation

		Measures to improve implementation						
		Training users	Monitoring imple-mentation	Monitoring symptom control & side effects	Clinic-wide standar-dization of opioid therapy	Open / uncom-plicated communi-cation	Positive error culture	Joint ward rounds
**Kruskal-Wallis H-Test (df = 2)**	H	2.945	2.205	3.031	4.624	3.127	6.505	2.822
	p	0.229	0.332	0.220	0.099	0.209	0.03	0.244
**Post-Hoc-Dunn-Test**								
General hospital PCS (prescription) vs. general hospital PCS (recom-mendation)	z						10.839	
	p						0.015	
General hospital PCS (prescription) vs. university hospital PCS (recommendation)	z						10.875	
	p						0.033	
General hospital PCS (recom-mendation) vs. university hospital PCS (recom-mendation)	z						-0.036	
	p						0.993	

* significant on a level of p < .05 (two-sided), ** significant on a level of p < .01 (two-sided),
