# Supplementary material for: Challenges in opioid therapy implementation: national survey of palliative care consultation services
Source: BMC Palliat Care. 2025 Oct 20;24:262. doi: 10.1186/s12904-025-01921-0 (PMC12539157; doi:10.1186/s12904-025-01921-0)
Supplement: Supplementary file 4 — Additional file 4: Content analysis of answers to open ended questions. Detailed description of methods and results of content analysis of open-ended answers; including original data. [file 12904_2025_1921_MOESM4_ESM.pdf]

Additional File 4: Content analysis of answers to open ended questions

Results

Tab. 4-1: Results of content analysis of open-ended questions on topics and situations of particular concern

Sub-category	Translations of original responses (from German)	Original responses in German
**Main category: Areas for improvement**		
**Unsatisfactory implementation of PRN medication**<br>Description: The administration of PRN (as-needed) strong opioids is often suboptimal, with reported errors in timing, frequency, and dosing. Additionally, insufficient preparation for specific situations, such as prior to physical exertion or diagnostic procedures, was noted.	• While the implementation of baseline medication usually works well, PRN medication practices require improvement.<br>• Uncertain Use of PRN Medications.<br>• Common errors in PRN dosing, especially regarding timing and frequency.<br>• PRN medication administration is often poorly executed on general wards, especially before physical exertion or examinations.<br>• Challenges in implementing PRN doses on the treating ward.<br>• Adjustment of sustained-release doses or perfusor flow rates based on the amount of PRN doses given.<br>• Clear communication of PRN dosing instructions to ward staff is needed.	• Die Umsetzung von Basismedikation funktioniert in der Regel gut. Die Umsetzung von der Bedarfsmedikation ist verbesserungswürdig.<br>• unsicherer Umgang mit Bedarfsmedikationen<br>• Bedarfgaben für die Stationen verständlich machen<br>• Fehler bei der Bedarfsdosierung va Abstand und Häufigkeit<br>• Anwendung von Bedarfsmedikation wird häufig auf Normalstationen nicht gut umgesetzt, insbesondere z.B. vor Belastungen oder vor Untersuchungen<br>• Umsetzung der Bedarfsgaben auf der behandelnden Station<br>• Anpassung der ret Dosis bzw Perfusorlaufrate an Hand der Menge der Bedarfsgaben
**Medication on discharge**<br>Description: Challenges in ensuring continuity of opioid prescriptions upon discharge, e.g. reluctance or inability to issue opioid prescriptions on wards or outpatient services (e.g. weekends)or change of application close to discharge.	• Continuation of prescriptions for patients discharged to home care is often interrupted (e.g., weekends, midweek afternoons, holidays). Not all colleagues or department heads are prepared to issue narcotic prescriptions (BTM).<br>• Transition from inpatient to outpatient care.<br>• Transitioning from subcutaneous (s.c.) or intravenous (i.v.) opioids shortly before patient discharge.<br>• Improvements are needed in BTM prescription practices within discharge management.	• Weiterverordnung bei Entlassung in die Häuslichkeit und der HA kann nicht nahtlos weiter verordnen (WoE, Mi-Nachmittag, Urlaub, ...). Nicht alle Kollegen, Klinikleiter halten BTM-Rezepte vor.<br>• Übergang Klinik-ambul. Bereich<br>• Umstellung von s.c. oder i.v. kurz vor der Entlasssung der Patient:innen<br>• Verbesserung der BtM Verschreibung im Entlassmanagement
**Inappropriate opioid use**<br>Description: Instances of inappropriate opioid use in situations when guidelines recommend alternative drugs or there is no indication at all.	• Cases of euthanasia with opioids carried out under pressure for free beds by attending physicians.<br>• Acceleration of the dying process.<br>• Repeated use of high opioid doses during the terminal phase without clear indication, coupled with resistance to advice.	• All die Fälle mit aktiver Sterbehilfe durch Opioide durch Primärbehandler unter Bettendruck<br>• Beschleunigung der Sterbephase<br>• Immer wieder hohe Opiatdosen in Sterbephase ohne klare Indikation, hier auch Beratungsresistenz
**Unsatisfactory symptom assessment**<br>Description: Inadequate symptom assessment on wards was identified as a factor delaying therapy optimization.	• Symptom assessment on other wards is often insufficient.<br>• When initiating strong opioids, thorough symptom assessment by the ward is essential to better evaluate the therapy's effectiveness. This enables quicker dose adjustments.	• Symptomassessment auf anderen Stationen nicht immer suffizient.<br>• Bei Start von starken Opioiden, sollte eine ausführliche Symptomassessment seitens der Station stattfinden, um die Wirkung der Therapie besser beurteilen zu können. Dies ermöglicht eine schnellere Dosisanpassung.
**Errors in opioid rotation:**<br>Description: Dosing problems due to lack of knowledge regarding opioid equivalence doses.	• Lack of knowledge about opioid equivalence doses on wards.	• Fehlende Kenntnisse bei Äquivalenzdosen von Opiaten auf den Stationen.

Sub-category	Translations of original responses (from German)	Original responses in German
**Errors in use of perfusors**<br>Description: General ward staff often encounter difficulties in managing opioid perfusors effectively	• Better handling of opioid perfusors on general wards to prioritize patient-centered care.<br>• System limitations in clinical information systems (CIS), e.g., perfusors can only be prescribed in ml/h rather than mg/h, among other issues.	• Verbesserte Umgang im Umgang mit Opioidperfusoren auf Normalstationen im Sinne der Patient:innenorientierung<br>• Fehlerquelle KIS (Perfusoren können nicht in mg/h, sondern nur in ml/h angeordnet werden uvm)
**Main category: Systemic and educational underlying factors**		
**Inexperience and lack of knowledge**<br>Description: Deficiencies in staff knowledge and experience were frequently cited as contributing to inappropriate opioid use. Both overly cautious and excessively liberal practices were reported.	• Carelessness among colleagues handling strong medications as if they were sweets.<br>• Persistent myths among professionals, family members, and patients that opioids accelerate or induce death.<br>• Underestimation of the specific requirements for handling opioids.<br>• Lack of knowledge about opioid equivalence doses on wards.	• Die Ahnungsfreiheit der Kollegen, die mit potenten Medikamenten umgehen als wären es Bonbons.<br>• Anhaltender Mythos bei Fachleuten, Angehörigen und Patienten, Opioide würden Sterben beschleunigen oder einleiten.<br>• Unterschätzung der Besonderheiten im Umgang mit Opioiden<br>• Fehlende Kenntnisse bei Äquivalenzdosen von Opiaten auf den Stationen.
**Resistance to training / advice**<br>Description: From the perspective of PCCS some staff and leaders in attending wards should be more open to advice and acknowledge the need for training.	• Department heads show little openness to existing training opportunities.<br>• A significant concern is colleagues who are "very resistant to advice" in their handling of opioids, which poses a danger as they fail to recognize the consequences of inappropriate opioid use for patients.<br>• Repeated use of high opioid doses during the terminal phase without clear indication, coupled with resistance to advice.	• Fehlende Offenheit der Chefärzte für Schulungsangebote, die es gibt<br>• Mich beschäftigt v.a., dass manche Kollegen im Umgang mit Opioiden "sehr beratungsresistent" sind. Das halte ich für gefährlich, da sie sich der Konsequenzen für Patienten bei nicht-indiziertem Einsatz von Opioiden nicht bewusst sind.<br>• Immer wieder hohe Opiatdosen in Sterbephase ohne klare Indikation, hier auch Beratungsresistenz
**Heterogeneous implementation across departments**<br>Description: The adoption of opioid recommendations and quality of collaboration varies greatly from department to department. Implementation was particularly limited in departments that are less frequently visited wards.	• Inconsistent adherence to recommendations across departments. The main issue is a lack of consideration of recommendations rather than incorrect implementation.<br>• Departments visited less frequently (e.g., cardiology, thoracic surgery, intensive care units) are the most hesitant to implement recommendations.<br>• Recommendations and orders regarding opioid use vary based on time constraints and relationships with treating physicians.	• sehr heterogene Umsetzung von Empfehlungen nach Fachabteilung. Problem ist eher nicht Beachtung der Empfehlungen als falsche Umsetzung.<br>• In meiner Erfahrung sind die Abteilungen, die wir am seltensten besuchen (u.a. Kardiologie, Thoraxchirurgie, Intensivstationen), am zögerlichsten bei der Umsetzung unserer Empfehlungen.<br>• Wir machen beides, Opioide Empfehlungen und Anordnungen je nach Zeit und Verhältnis zu den PrimärbehandlerInnen.
**Limited opioid availability**<br>Description: Restrictions in the availability and variety of opioids in stock at both hospital and ward levels were reported.	• Restrictions in opioid selection due to stock limitations in the hospital pharmacy.<br>• Ensure that commonly used low-dose formulations of 2–3 opioids are available on every ward in various administration forms.	• Begrenzung in Opioidauswahl durch Bevorratung in der Klinikapotheke<br>• Niedrige Dosierungen der gängigen 2-3 Opioide auf jeder Station vorrätig haben und in verschiedenen Applikationsformen.
**Staff turnover**<br>Description: Staff turnover complicates individualized and adequate opioid therapy.	• While expert standards serve as a good foundation, individualized concepts are challenging to implement due to frequent personnel turnover in both nursing and medical staff.	• gute Expertenstandards sind gute Grundlage, aber individualisierte Konzepte wegen fehlender Kontinuität bei den Hauptbehandlern (häufige Personalwechsel in Pflege und ärztlich) schwer umzusetzen;
**Limitations of clinical information system**<br>Description: Limitations in the clinical information system, such as restricted options for specifying doses and other parameters, were identified as impeding the effective communication of opioid recommendations.	• System limitations in clinical information systems (CIS), e.g., perfusors can only be prescribed in ml/h rather than mg/h, among other issues.	• Fehlerquelle KIS (Perfusoren können nicht in mg/h, sondern nur in ml/h angeordnet werden uvm)

Tab. 4-2: Results of content analysis of open-ended questions on measures to improve adherence to opioid recommendations

Sub-category	Translations of original responses (from German)	Original responses
**Main category: Measures in attending wards**		
**Education and training**<br>Description: Various suggestions for educational initiatives aimed at improving the knowledge and confidence of medical and nursing staff in palliative care and opioid therapy are suggested. The need to include palliative topics in medical curricula and certification requirements is also highlighted.	• Structured training programs at the start of employment in the hospital—for physicians and nurses—with periodic updates and hospital-wide standardization.<br>• Increased training.<br>• Additional educational opportunities to reduce existing fears and prejudices among patients, relatives, and even doctors.<br>• Regular mandatory training similar to "Last Aid" courses, analogous to resuscitation training.<br>• Mandatory training on pain management and general palliative care.<br>• Low-threshold educational offerings.<br>• Fixed training sessions multiple times a year focusing on safe, evidence-based pharmacotherapy for frail and palliative patients—not just on opioids but also on other important medications for symptom control.<br>• More teaching time dedicated to palliative medicine topics in medical education.<br>• Mandatory further training on pain management and palliative medicine topics (e.g., opioids and communication) as part of board certification requirements across nearly all clinical disciplines.<br>• End-of-life (EoL) courses, including pain management and palliative care, as part of annual mandatory training for all medical and nursing staff—similar to resuscitation courses.<br>• Ward-specific training, both in practical application and in addressing fears.<br>• More nurses with training in palliative care.	• Strukturiertes Schulungsprogramm bei Arbeitsbeginn in der Klinik - für Ärzte und Pflege. Turnusmäßige Erneuerung der Kenntnisse und klinikweite Synchronisierung.<br>• Mehr Schulung.<br>• weitere Bildungsangebote um bestehende Ängste und Vorurteile bei Pat, Angehörigen aber auch Ärzten abzubauen<br>• Regelmäßige Pflicht-Schulungen in ähnlich "Letzte Hilfe Kurse" analog Reanimationsschulungen.<br>• Pflichtschulung zur Schmerztherapie/allgemeine Palliativversorgung.<br>• niedrigschwelliges Fortbildungsangebot.<br>• Mehr Pflegekräfte mit Ausbildung palliative care<br>• Mehrmals im Jahr feste Schulungen zur sicheren, evidenzbasierten Pharmakotherapie bei gebrechlichen, palliativen und Patienten, und nicht nur über Opioide, sondern andere wichtigen Medikamente für die Symptomkontrolle<br>• mehr Zeiteinheiten für PAll Med Themen in der studentischen Lehre; mehr Pflicht-Weiterbildungen zu Schmerz und Pall Med Themen z B Opioide und Kommunikation für Facharztprüfungen in fast allen klinischen Fächern,<br>• EoL-Kurse d.h. Schmerztherapie und Palliativmedizin als Teil jährlicher verpflichtender Schulungen allen Arzt-&Pflegepersonals zum Ende des Lebens - so wie es Rea-Kurse gibt.<br>• Schulungen der Station sowohl in der Anwendung als auch Ängste nehmen
**Internal support processes**<br>Description: Enhancing support for less experienced staff through mentorship from specialists and incorporating palliative care nurses as key resources on all wards.	• Greater support for junior physicians from experienced specialists.<br>• Palliative care nurses on all wards to act as multipliers.	• Mehr Unterstützung der jungen Kollegen durch erfahrene Fachärzte.<br>• Palliativpflegekräfte auf allen Stationen als Multiplikator:innen.
**Main category: Measures in PCCS**		
**Independent prescribing authority**<br>Description: Empowering the palliative care team to independently initiate opioid therapy, ensuring timely and effective pain management without unnecessary delays.	• Granting the palliative care team autonomy to initiate opioid therapy independently ("opioid authority")	• Erlaubnis zum selbstständigen Ansetzen von Opioiden durch den Palliativdienst ("Opioidhoheit").
**Educational role as part of self-image**<br>Description: Education in attending wards as part of the work of PCCS	• The palliative care team also serves an educational role for staff.<br>• Education / further training in an integrated-consultative model enhances security for patients.	• Palliativdienst hat auch Bildungsfunktion für Mitarbeiter!<br>• Edukation/FB für ein gemischt integrativ- konsiliarisches Modell eines Pall-Dienstes bietet besser Sicherheit für Patient*innen

Sub-category	Translations of original responses (from German)	Original responses
**Main category: Measures in collaboration of attending wards and PCCS**		
**Monitoring by palliative care services**<br>Description: Suggestions regarding the role of the PCCS in providing regular and frequent bedside visits, early detection of errors, and continuous support for patients and staff.	• The palliative medical team (PMD) sees all involved patients (presented for consultation) daily, allowing discrepancies and errors to be identified and communicated promptly.<br>• Advantages of palliative complex therapy, with daily bedside visits and 24/7 availability of a palliative care team on-call. Education and further training in an integrated-consultative model enhances security for patients.<br>• More frequent involvement of palliative care teams to manage opioid therapy.	• Unser PMD sieht alle eingebundenen (per Konsil vorgestellten) Patienten täglich. Somit können Unstimmigkeiten und Fehler rasch erkannt und kommuniziert werden.<br>• Vorteil bietet Palliativkomplextherapie mit täglichen zusätzlichen Besuchen am Bett und 24/7 Verfügbarkeit eines Pall Dienstes in Rufbereitschaft, Edukation/FB für ein gemischt integrativ- konsiliarisches Modell eines Pall-Dienstes bietet besser Sicherheit für Patient*innen<br>• Häufigerer Einsatz der Palliativdienste zur Steuerung der Opioidtherapie.
**Integrated / interdisciplinary care**<br>Description: Joint bedside visits attending teams and PCCS, to enhance knowledge transfer and patient safety-	• Education / further training within an integrated-consultative model enhances security for patients.<br>• Greater focus on interdisciplinary bedside exchange—physicians, nursing staff, and other disciplines—with more continuity in patient care teams.<br>• Enhanced communication	• Edukation/FB für ein gemischt integrativ- konsiliarisches Modell eines Pall-Dienstes bietet besser Sicherheit für Patient*innen<br>• mehr Fokus auf gemeinsamen Austausch am Bett ärztlich, pflegerisch, weitere Disziplinen- mehr Kontinuität bei den Behandlern.<br>• Mehr Kommunikation
**Positive error culture**<br>Description: Fostering non-punitive approach to error reporting.	• Open communication and a positive error culture throughout the hospital.	• Offene Kommunikation und positive Fehlerkultur an der gesamten Klinik
**Uniform protocols / standard operating procedures in opioid therapy**<br>Description: The establishment of uniform protocols and standard operating procedures (SOPs) for palliative care, including access to standardized medication sets and clear pain management guidelines.	• Immediate establishment of a unified "SOP for the terminal phase" across the entire hospital, with standard "palliative medications" available on every ward.<br>• Recommendations for a standardized pain management concept for palliative patients, e.g., in a card format.	• Sofortige Etablierung von einheitlicher "SOP Sterbehase" fürs gesamte Haus mit einheitlicher "Palli-Medikation" auf jeder Station verfügbar<br>• Empfehlungen für Schmerzkonzept für Palliativpatienten z.B. Kartenformat.
**Timely integration of palliative care**<br>Description: The importance of involving palliative care teams early in the treatment process, rather than as a last resort. This emphasizes the need to overcome misconceptions about palliative care being solely for end-of-life situations.	• Early involvement of the palliative care team. Too often, consultations occur only when patients have significant decline or limited tumor-directed treatment options.<br>• Palliative care must not be perceived as a "death service."<br>• Changing the perception of "palliative care" as purely end-of-life care, especially among colleagues, would help many patients much earlier.	• Palliativdienst frühzeitig anzubinden!! Zu oft konsiliarische Anfrage bei zunehmender AZ-Minderung der Pat. und eingeschränkten tumorgerichteten Therapieoptionen<br>• .Nicht als "Sterbedienst" empfunden zu werden!<br>• Dass "palliativ" das Image reiner Sterbebegleitung verliert - und zwar insbesondere in den Köpfen der Kollegen. Damit wäre sehr vielen Patienten sehr viel eher geholfen
**Main category: hospital-wide / system**		
**Staffing and resources**<br>Description: Need for increased staffing and time in generalist and specialist palliative care.	• Increased personnel and time resources.<br>• More staff resources.<br>• More nurses with training in palliative care.<br>• Most importantly: public education and funding for “speaking medicine”, both inpatient and outpatient.	• mehr personelle / zeitliche Ressourcen<br>• Mehr Personalressourcen<br>• Mehr Pflegekräfte mit Ausbildung palliative care<br>• Das Wichtigste: Aufklärung: Finanzierung von gesprochener Medizin stationär und ambulant
